# Adult Diffuse Low-Grade Gliomas: 35-Year Experience at the Nancy France Neurooncology Unit

**DOI:** 10.3389/fonc.2020.574679

**Published:** 2020-10-28

**Authors:** Tiphaine Obara, Marie Blonski, Cyril Brzenczek, Sophie Mézières, Yann Gaudeau, Celso Pouget, Guillaume Gauchotte, Antoine Verger, Guillaume Vogin, Jean-Marie Moureaux, Hugues Duffau, Fabien Rech, Luc Taillandier

**Affiliations:** ^1^Centre de Recherche en Automatique Nancy France - UMR 7039 – BioSiS Department, Faculty of Medicine, Université de Lorraine, Vandoeuvre-lès-Nancy, France; ^2^Neurology Departement, Neurooncology Unit, CHRU, Nancy, France; ^3^Department of Mathematics, Elie Cartan Institute, Nancy, France; ^4^INRIA Biology, Genetics and Statistics, Nancy, France; ^5^Department of Pathology, CHRU, Nancy, France; ^6^Centre de Ressources Biologiques, BB-0033-00035, CHRU Nancy, France; ^7^Department of Nuclear Medicine and Nancyclotep Imaging Platform, CHRU Nancy, France; ^8^IADI, INSERM U1254, Lorraine University, Vandoeuvre-lès-Nancy, France; ^9^UMR 7365 CNRS, IMoPA Biopole Lorraine University Faculty of Medicine, Université de Lorraine, Vandoeuvre-lès-Nancy, France; ^10^Department of Radiation Therapy, Baclese Radiation Therapy Centre, Esch/Alzette, Luxembourg; ^11^Department of Neurosurgery, Montpellier University Medical Center, Gui de Chauliac Hospital, Montpellier, France; ^12^Team “Plasticity of Central Nervous System, Stem Cells and Glial Tumors”, U1051 Laboratory, National Institute for Health and Medical Research (INSERM), Institute for Neurosciences of Montpellier, Montpellier University Medical Center, Montpellier, France; ^13^Department of Neurosurgery, CHRU, Nancy, France

**Keywords:** diffuse low-grade glioma, prognosis, survival, quality of life, surgery, chemotherapy, radiation therapy

## Abstract

**Background:**

To report survival, spontaneous prognostic factors, and treatment efficacy in a French monocentric cohort of diffuse low-grade glioma (DLGG) patients over 35 years of follow-up.

**Methods:**

A monocentric retrospective study of 339 patients diagnosed with a new DLGG between 01/01/1982 and 01/01/2017 was created. Inclusion criteria were patient age ≥18 years at diagnosis and histological diagnosis of WHO grade II glioma (according to 1993, 2007, and 2016 WHO classifications). The survival parameters were estimated using the Kaplan-Meier method with a 95% confidence interval. Differences in survival were tested for statistical significance by the log-rank test. Factors were considered significant when *p* ≤ 0.1 and *p* ≤ 0.05 in the univariate and multivariate analyses, respectively.

**Results:**

A total of 339 patients were included with a median follow-up of 8.7 years. The Kaplan-Meier median overall survival was 15.7 years. At the time of radiological diagnosis, Karnofsky Performance Status score and initial tumor volume were significant independent prognostic factors. Oncological prognostic factors were the extent of resection for patients who underwent surgery and the timing of radiotherapy for those concerned. In this study, patients who had delayed radiotherapy (provided remaining low grade) did not have worse survival compared with patients who had early radiotherapy. The functional capabilities of the patients were preserved enough so that they could remain independent during at least three quarters of the follow-up.

**Conclusion:**

This large monocentric series spread over a long time clarifies the effects of different therapeutic strategies and their combination in the management of DLGG.

## Highlights

This article relates a long period (35 years) French neurooncology center experience concerning a cohort of DLGG patients. This is the first article describing an overview. The number of patients (339) is substantial for a monocentric approach. Our results confirm the importance of awake surgery with cortico-subcortical intraoperative stimulations, and identify prognostic factors consistent with other series already published. The analysis of overall survival was made by integrating patient's quality of life.

## Introduction

Diffuse low-grade gliomas are rare tumors (about 15% of gliomas) in young and middle-age adults (median age at diagnosis is around 40 years) at the interface of neuroscience and oncology (1–3). The conventional therapeutic approach with surgery, radiotherapy, alkylating agent–based chemotherapy has drastically changed in the last 15 years and allowed—a unique fact in oncology—doubling the median survival while preserving quality of life (QoL) without new therapeutic tools but just by progressing in the so-called onco-functional balance analysis (4). These advances are primarily related to awake functional surgery and its articulation with chemotherapy (5). Radiotherapy remains a subject of discussions concerning timing, optimal dose–volume distribution, and association or not with chemotherapy and its potential toxicity (6). Many questions continue to feed the debates, essentially related to the slow evolution of the heterogeneous disease, the succession different therapeutic steps adapted to each patient, and the limits of evidence-based medicine for this pathological profile (7). As a result, analysis of databases remain relevant (7). We report results from a single French center over a period of more than 35 years. We analyze spontaneous and therapeutic prognostic factors and survival for 339 patients with DLGG.

## Methods

### Patients Selection

The patients selected were extracted from the neurooncology unit of the Nancy France University Hospital database containing more than 400 consecutive patients diagnosed with DLGG according to various WHO classifications over time between 01/01/1982 and 01/01/2017. To be included, patients had to fulfill the following criteria: age ≥18 years at radiological diagnosis and pathological diagnosis of WHO grade II gliomas. Exclusion criteria were a medical follow-up not entirely carried out in Nancy, gliomatosis defined as involvement of 3 or more lobes except fronto-temporo-insular locations, and death not related to the tumor. All living patients provided written informed consent regarding the use of their data.

### Definitions and Concepts

Here, we introduce concepts and definitions used in the following parts and helpful for the reader’s understanding. We have defined the “delayed treated” group as patients who didn’t have any treatment within the 2 years following the radiological diagnosis, and the “early treated” group received at least a first-line treatment in this interval of time. Several types of first-line treatment were considered, including surgery alone (S), chemotherapy alone (CT), radiotherapy alone (RT), surgery + adjuvant chemotherapy (S+CT), surgery + adjuvant radiotherapy (S+RT), or chemotherapy + radiotherapy (CT+RT). Adjuvant treatments were administrated within the 3 months following the surgery or chemotherapy. Concerning surgery, gross total, subtotal, and partial resection are defined by no residual tumor volume, residue ≤10 and ≥10 cm^3^ on flair-weighted MRI, respectively. The choice of these three groups resulted from a previous study evaluating the role of radical resection in DLGG (8). Patients who underwent radiotherapy were divided into three groups according to the timing of irradiation; in the “early radiotherapy” group, patients had irradiation at first- or second-line treatment; in the “delayed radiotherapy” group, patients had irradiation at least after the third line; the last group included patients with radiotherapy after malignant transformation.

### Treatment Practice

Two modalities of chemotherapy were used: temozolomide (TMZ) or a combination of procarbazine + CCNU + vincristine (PCV). TMZ was used conventionally (150 then 250 mg/m2 orally, 5/28 days). The duration was variable over time. In general, treatment was prolonged as long as the patient responded subject to acceptable tolerance. The majority received from 12 to 24 cycles. We also used standard PCV with a majority of patients who received 3 to 6 cycles. Procarbazine was administered at the dose of 60 mg/m^2^ orally per day on days 8 through 21 of each cycle, CCNU at a dose of 110 mg/m^2^ on day 1 of each cycle, and vincristine at a dose of 1.4 mg/m^2^ with a maximum dose of 2.0 mg administered intravenously on days 8 and 29 of each cycle. The cycle length was 6 to 8 weeks.

The modalities of radiotherapy were conventional. Patients received 54 to 59.4/60 Gy with standard 1.8 to 2 Gy each (prescribed to the isocenter) over a period of 6 weeks.

### Data Collection

The data used in this study were recovered from the 339 patients having met inclusion criteria. Clinical data included patient age, sex, date plus type of first symptoms (seizure, neurological trouble, headache) and Karnofsky Performance Status (KPS) score at diagnosis and during the follow up. Imaging data included location, number of involved lobes, contrast enhancement, and volume. The volumetric quantification was performed using the three diameters technique (9) at the beginning (films) and then using the manual segmentation under OsiriX software (digital imaging) (10). All volume evaluations were performed exclusively by two physicians (MB, LT) using the data provided by the following MRI sequences: T1-weighted before and after gadolinium injection, T2-weighted and fluid attenuation inversion recovery (FLAIR) sequences for the volumes. Concerning treatment, the following data were collected: type of surgery (conventional or functional-guided surgery), extent of resection, and the chemotherapeutic regimen (drugs, number of cycles). About pathology, diagnoses were made on the different WHO classifications (11–13). The main biomolecular variables KI67 index, 1p19q codeletion, and IDH mutation were also recovered if available. Malignant transformation was considered when histologically proven or when nodular and intense enhancement.

### Statistical Analysis

All data analyses were performed using R version 1.1. For descriptive statistics, we used numbers and percentages; for qualitative ones, variables; and for quantitative ones, medians and ranges. The starting point chosen for our analyses was the radiological diagnosis. Survival was calculated as the time from the radiological diagnosis until death. Survival curves were estimated using the Kaplan-Meier method with a 95% confidence interval and differences tested by the log-rank test. To check the assumption of proportional hazards, we used graphical evaluation. To assess prognostic factors, univariate analysis was first performed using log-rank tests for qualitative variables and univariate Cox models for quantitative ones. Prognostic factors with *p* value ≤ 0.1 were considered as candidates for the multivariate Cox regression analysis. In the final analysis, only factors with *p* value ≤ 0.05 were considered statistically significant.

## Results

### Patient Characteristics

Summary patient characteristics for the data sets used in this analysis are reported in **Table 1**. A total of 339 patients diagnosed were included. At radiological diagnosis, the median age was 38 years with a large part of patients diagnosed between 34 and 60 years. Epilepsy was the most common symptom leading to let on the lesion (83.2%), and headache and neurological troubles revealed the tumor in 8.6% and 4.1% of cases, respectively. Incidental detection represented 4% of the cases. Concerning the radiological characteristics, the frontal lobe was invaded in 67.4% of cases, and the temporal lobe was invaded in 25.3% of the cases. Moreover, 33.1% of patients presented a tumor with multiple lobes affected. We noted a contrast enhancement for 25.1% of the cases, usually patchy (16.8%) and less often nodular (5.3%) or ring like (2.4%), and pathological examination confirmed the WHO II grade in all of these cases. Median volume on diagnosis MRI was 49 cm^3^. As regards the histological type, the biomolecular data as IDH and 1p19q status were unavailable for 48% and 46.4% of patients; 58.7% of tumors were identified as oligodendroglioma, 25.4% as astrocytomas, and 15.9% were oligoastrocytoma.

**Table 1:** Summary of epidemiological characteristics at radiological and histological diagnosis.

	Parameter	No.	%
**Patient sex**	female male	151 188	44.5 55.5
**Age in years**	]18;34] ]34-60] ]60-80]	181 130 28	53.4 38.3 8.3
**Median age in years (range)**		38 (18-80)	
**Symptom**	epilepsy neurological trouble headache incidental finding	282 14 29 14	83.2 4.1 8.6 4.1
**KPS score median (range)**		100% (50%-100%)	
**Tumor location**	frontal frontal + parietal frontal + temporal frontal + insular temporal temporal + insular parietal occipital multifocal basal ganglia	171 14 10 34 32 44 16 8 6 4	50.4 4.1 2.9 10.0 9.4 13.0 4.7 2.4 1.8 1.2
**Number of lobes involved**	1 2 ≥ 3	227 102 10	66,9 30 3.1
**Tumor side**	right left bilateral	156 174 9	46.0 51.3 2.6
**Contrast enhancement**	no patchy nodular ring NA	254 57 18 8 2	74.9 16.8 5.3 2.4 0.6
**Tumor volume (in cm^3^)**	** **< 49 ≥ 49 NA	136 134 69	40.1 39.5 20.4
**Biomolecular**	IDH mutated IDH nonmutated NA codeletion 1p19q noncodeletion 1p19q NA	128 33 178 64 107 168	37.8 9.7 52.5 18.8 31.6 49.6
**Histological type**	Oligodendroglioma Astrocytoma Oligoastrocytoma	199 86 54	58.7 25.4 15.9

NA, data no available at the time of the study.

### Therapeutic Strategy

**Figure 1:**
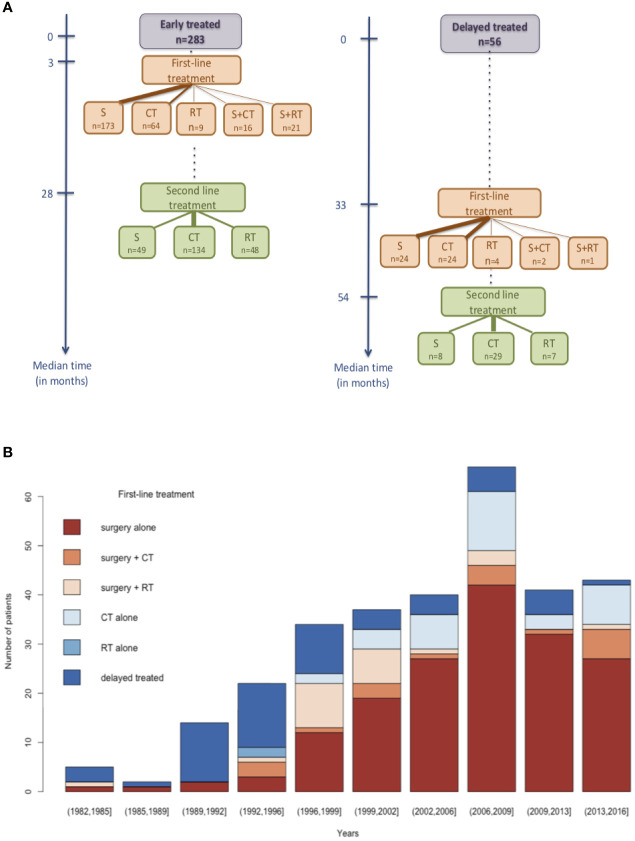
First-line therapeutic strategy and its evolution over time. **(A)** The median time delay is presented for both groups: patients who were early treated and delayed treated. The width of the arrows is proportional to the percentage of patients that have received the different treatments. **(B)** Change of first-line therapeutic strategy over time. S, surgery; CT, chemotherapy; RT, radiotherapy.

As summarized in **Figure 1A**, among the 339 patients with a DLGG radiological diagnosis, the initial strategy consisted of early treatment for 283 patients (83.5%), and close monitoring was preferred for 56 patients (16.5%). The delayed treatment was preferred for patients who had better prognosis regarding their clinical and radiological data (low volume, no contrast, low kinetic profile). In the early treated group, a median of 3 months separated the radiological diagnosis from the first-line treatment (surgery, chemotherapy, rarely radiotherapy or combined treatments), and it was 33 months in the monitoring group. The time between the two therapeutic sequences did not differ between the groups (25 vs. 21 months). The first-line treatment consisted of surgery alone for a large number of patients (61%) in the “early treated” group, and chemotherapy and surgery were indifferently performed in the “delayed treated” group. As shown in **Figure 1B**, the first-line treatment has constantly evolved over time, and surgery has become the primary first-line treatment (from 33% in 1989–1996 to 62% in the last period 2013–2016). Adding all the patients who had at least a surgery in their first-line treatment (S alone, S+CT, S+RT), the proportion of patients who were early operated reaches 70%. For those not involved in the surgery group, chemotherapy alone largely dominates. **Table 2** summarizes the characteristics of oncological parameters for the 338 patients treated at the grade II stage (1 patient recently included had a biopsy without any treatment). Regardless of the initial strategy, the first-line treatment mostly consisted of resection alone (58.3%) and, less often, chemotherapy alone (25.4%), while radiotherapy alone was marginal (3.3%). When surgery was decided, the resection was performed according to functional awake surgery in 43.4% of the cases. Gross total resection and subtotal resection were achieved in 20% and 61.8% of cases, respectively. When patients were treated by chemotherapy, the first regimens consisted predominantly of TMZ (77.8%) and PCV (17.2%) protocol, whereas Fotemustine (FTM) and Carmustine (BCNU) were marginally administered. Regarding radiotherapy, the proportion of patients in the “early radiotherapy,” “delayed radiotherapy,” and “after maligant transformation” groups were respectively 23.9%, 57.5%, and 18.6%.

**Table 2:** Oncological treatment parameters.

Parameter	Number (%)
**Type of first-line treatment**	***n*=338**
surgery	
alone	197 (58.3)
+ CT	18 (5.3)
+ RT	22 (6.5)
CT	
alone	84 (25.4)
+ RT	4 (1.2)
RT	
alone	13 (3.3)
**Type of first surgery**	***n*=237**
conventional	134 (56.6)
functional	103 (43.4)
**Postoperative tumor volume**	***n*=237**
≥ 10 cm^3^	31 (18.2)
< 10 cm^3^	105 (61.8)
no residual tumor	34 (20)
NA	67
**Type of first chemotherapy**	***n*=274**
TMZ	213 (77.8)
PCV	47 (17.2)
FTM	9 (3.2)
BCNU	5 (1.8)
**Moment of radiotherapy**	***n*=167**
early	40 (23.9)
delayed	96 (57.5)
after anaplastic transformation	31 (18.6)

CT, chemotherapy; RT, radiotherapy; TMZ, Temozolomide; PCV, procarbazine-CCNU-vincristine; FTM, fotemustine; BCNU, carmustine; NA, data non available at the time of the study.

### Prognostic Factors and Survival Outcome

#### Overall Survival

**Figure 2:**
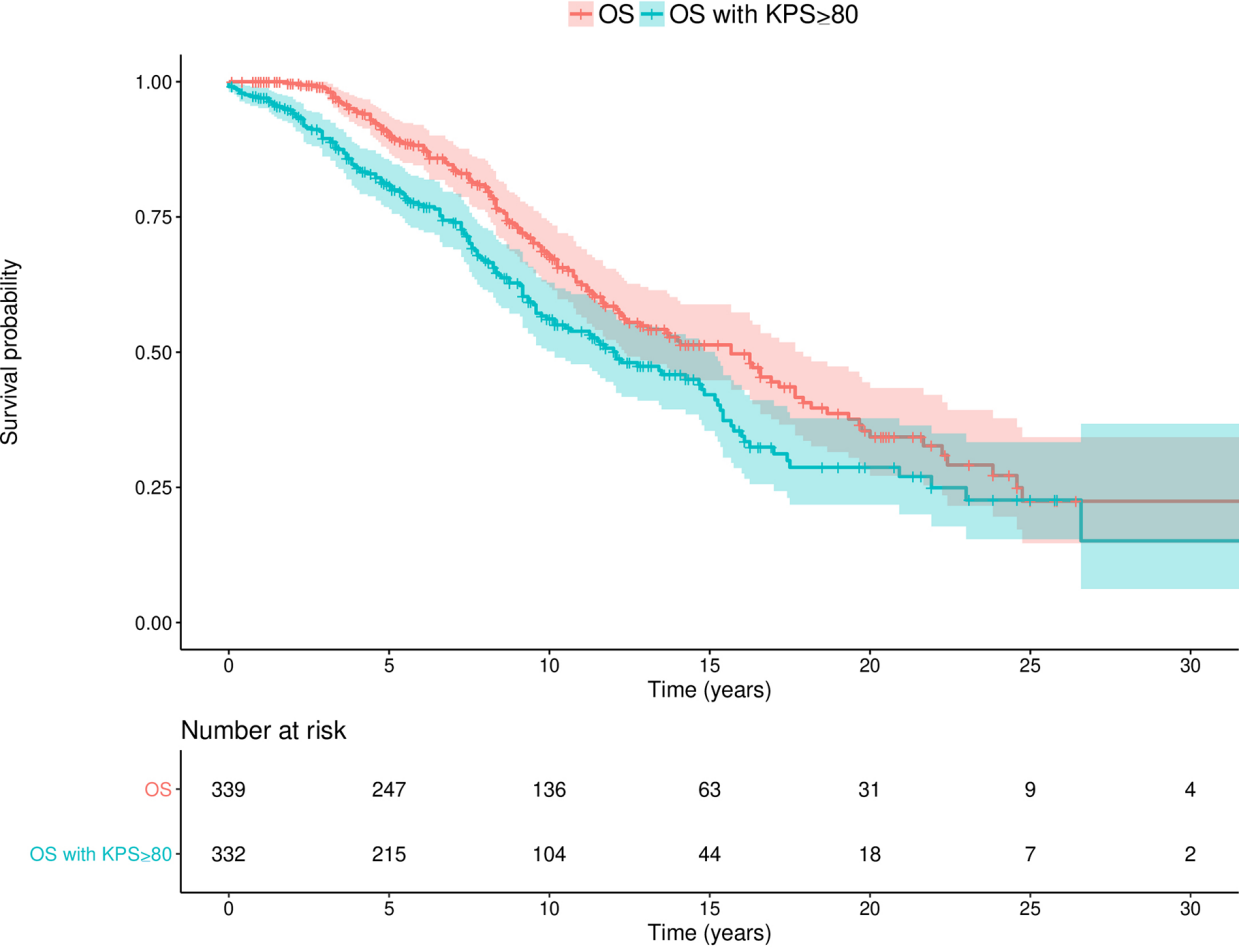
Overall survival since radiological diagnosis. Kaplan-Meier estimate curves (with 95% CI) showing both the OS and the OS with a KPS score above or equal to 80.. OS, Overall survival; KPS, Karnofsky Performance Status.

Kaplan-Meier OS estimates are displayed in **Figure 2**. The median OS time since radiological diagnosis was 15.7 years (95% CI: 12.3-17.9), and the median time with a KPS score above or equal to 80 was 12.2 years (95% CI: 10.2-15.2). Note that we could not assess the initial KPS score for 7 patients because they presented an altered QoL not related to their cerebral tumor.

During the follow-up, 142 (41.9 %) patients died at a mean delay of 9.6 years (median at 8.7) after radiological diagnosis. The mean observed survival for the 197 patients alive at the last follow-up since radiological diagnosis was 9.6 years (median at 8.2). Malignant transformation occurred in 158 (46.6%) cases at a mean delay of 7.6 years (median at 6.9) since radiological diagnosis. The median delay between malignant transformation and death was 1.4 years.

Finally, the OS rates at 5, 10, and 15 years were respectively 89.6%, 69.8%, and 56.8%.

#### Spontaneous Prognostic Factors

**Figure 3:**
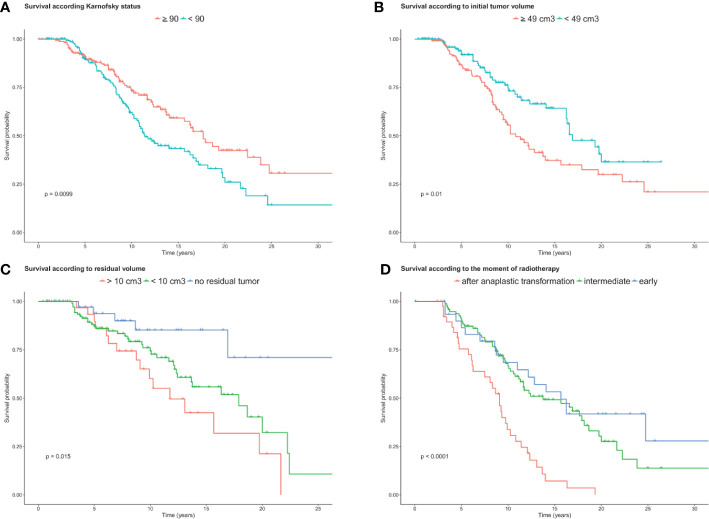
Overall survival curves. **(A)** Kaplan-Meier estimate curves showing the overall survival according to KPS (≤90 vs >90, *n*=339 patients). **(B)** Kaplan-Meier curves showing the overall survival according to the tumor volume at diagnosis (<49 cm^3^ vs. ≥49 cm^3^, *n*=339 patients). **(C)** Kaplan-Meier estimate curves according to postoperative residual volume (*n*=191 patients). **(D)** Kaplan-Meier estimate curves according to the moment of radiotherapy (*n*=167 patients).

Univariate and multivariate analyses are summarized in **Table 3**. The univariate analysis was performed for each pretreatment factor presented in **Table 1** and for oncological parameters presented in **Table 2**. At the time of radiological diagnosis, a priori independent prognostic factors were KPS score and initial volume (**Figure 3**). Other parameters, such as number of lobes involved and tumor location (single frontal location vs. other sites), were significant in univariate analysis only.

**Table 3:** Baseline demographic and oncological predictors (univariate and multivariate models).

	Univariate analysis		Multivariate analysis	
Factor	p-value	p-value	HR(95% CI)	Estimated median OS (years)
**Sex**	0.15			
**Age**	0.3			
**Symptoms**	0.6			
**KPS score**	0.01	0.03		
<90			1	8.75
≥90			0.48 (0.25-0.91)	16.25
**Main tumor location**	0.003	NS		
**Number of lobes involved**	<0.0001	NS		
**Tumor side**	0.56			
**Contrast enhancement**	0.5			
**Initial tumor volume**	0.01	0.05		
<49 cm^3^			1	16.9
≥49 cm^3^			1.72 (1.14-2.59)	10.8
**Histological type **	0.5			
**Type of first-line treatment**	0.2			
**Type of first-line surgery**	0.18			
**Postoperative volume**	0.015	0.015		
≥10 cm^3^			1	11.7
<10 cm^3^			0.65 (0.36-1.18)	17.9
no residual tumor			0.24 (0.08-0.66)	Not reached
**Type of first CT**	0.077	NS		
**Moment of RT**	<0.0001	<0.0001		
after anaplastic transformation			1	9
early			0.27 (0.14-0.50)	15.3
delayed			0.36 (0.23-0.56)	13.7

HR, hazard ratio; CI, confidence interval; OS, overall survival; RT, radiotherapy; CT, chemotherapy; NS, not significant.

#### Subgroup Analysis

In the subgroup analysis, prognostic factors were postoperative tumor volume for patients operated and moment of radiotherapy for those who had radiotherapy. We also assessed if the type of first chemotherapy could impact the survival.

##### Residual Volume

A total of 237 patients were resected with partial, subtotal, or gross total resection. The patients who underwent a complete resection had better survival than those who had partial or subtotal resection (median not reached, log-rank test: *p*=0.01). Kaplan Meier curves comparing OS of the residual volume are displayed in **Figure 3**.

##### Timing of Radiotherapy

Among the 339 patients, 167 received radiotherapy at different stages of their illness. The timing of irradiation was decided according to the symptomatology, histological, and/or radiological criteria. Considering the three groups (early, delayed, or after malignant transformation), we did not find a significant difference in survival between early and delayed radiotherapy (15.6 vs. 17.9 years, **Figure 3**), but the median OS survival was worse when patients were treated after malignant transformation (8.3 years, *p*<0.0001). Furthermore, among the 172 patients who have not (or not yet) been treated with radiotherapy at the end of the follow-up, 30 are dead (median survival at 8.3 years), and 142 were still alive with an estimated Kaplan-Meier OS of 22.4 years.

##### Type of First Chemotherapy

In this series, 274 patients were treated by chemotherapy during the follow-up. Before their tumor had progressed, 213 patients received TMZ, and PCV was administrated in 47 of them. Moreover, 5 received BCNU, and 9 received FTM. We compared survival according to the type of the first chosen chemotherapy (TMZ vs. PCV) and found better, but not significant, survival for patients treated by PCV chemotherapy especially for patients with an initial volume >49 cm^3^ (median at 17.2 vs. 10.2 years, *p*=0.065).

### Biomolecular Data

The IDH status was obtained for 175 (51.6%) patients of which 139 had an IDH mutation. The 1p19q codeletion status was obtained for 182 (53.6%) patients of which 64 had an 1p19q codeleted glioma. The estimated Kaplan-Meier OS was 18.6 years (95% CI: 13- not reached) for patients IDH mutated, 16.5 years (95% CI: 15.7- not reached) for patients 1p19q codeleted, 9.3 years (95% CI: 8.3- not reached) for patients with IDH mutation and without 1p19q codeletion and 15.9 years (95% CI: 11- not reached) for patients without IDH mutation nor 1p19q codeletion. Unfortunately, we were unable to interpret these results or perform more statistical analyses because the number of events in each subgroup was too small.

## Discussion

To our knowledge, this cohort of 339 patients included since 1982 is one of the largest and longest follow-up monocentric retrospective studies of DLGG. All patients were indeed followed from the diagnosis to the end of their illness in the same center with a common ambition of personalized medicine. The results are relevant because of the long median follow-up (close to the mean delay observed deaths), the low rate of those lost to follow-up (<5%), and the few missing data (except the biomolecular component). The study finds an estimated Kaplan-Meier OS close to 16 years. This OS estimation is better than an older retrospective series reported in the early 2000s with an estimated median OS at 6.4 years (14) or some more recent studies (15–21). Considering other monocentric series equivalent in number of patients, such as the Mayo Clinic (22), the results appear better in terms of median OS with 15.7 years versus 6.9 years despite the difficulty to compare populations because of the important median follow-up difference (13.6 vs. 8.7 years) and the disparity of initial populations in terms of histological types and extent of surgery.

QoL assessment was implicit during patient follow-up. We have formalized data (EORTC QLQC30 + BN 20) for a limited number of patients and, unfortunately, not always longitudinally. In the same way, neuropsychological data can indirectly reflect the QoL. It was systematically collected around surgical procedures and more randomly during the follow-up (especially for patients who were diagnosed more than 20 years ago). In this article reporting the follow-up of a homogeneous cohort over a long period, it nevertheless seemed essential to use a marker that could indirectly reflect functional evolution of patients and, therefore, their QoL even if, of course, this parameter remains too reductive to reflect the overall concept of QoL. So we decided to report the evolution of the KPS over time and more precisely the time of survival with a KPS score above or equal to 80. The estimated Kaplan-Meier was 12.2 years, signifying that the patients can have a normal life for the most part of their disease. With an overall survival of 15.7 years, we nevertheless note a 3.5-year differential, i.e., a survival with less than 80 KPS for a period of 3.5 years. Work in progress should allow us to better analyze events occurring in the last years of life.

Concerning the prognostic factors, consistent with the literature, our study confirms that, at the time of radiological diagnosis, KPS and volume at diagnosis are independent strong prognostic factors (23, 24). However, other factors such as age, contrast enhancement and histological type, described as strong prognostic factors in others studies, were not retrieved.

**KPS**. A significant difference was observed between the patients with a KPS <90 compared with those who had a KPS ≥90. This result is coherent because KPS alteration is essentially related to either comorbidities or a marked tumoral evolution with functional connectivity infiltration (25).

**Volume at Diagnosis**. We confirmed the importance of the volume in the prognosis with the choice of 2 classes (≤49 vs. >49 cm^3^). This factor has been described in several other studies as a poor factor prognosis (24). The number of involved lobes is a significant factor on univariate analysis but not on multivariate certainly due to the correlation with the tumor volume. We can indeed imagine that large volumes are correlated with the infiltration of the minimal common brain (26) with the impossibility of radical surgery related to a poor prognosis.

**Age**. Although age is considered as a strong prognostic factor in many studies (23, 27), we did not find significant difference in OS between three age classes (<34, 35–60 and >60). Age can be linked to a worse prognosis for various reasons: presence of intrinsically more pejorative tumor factors (including molecular factors) or an “under treatment” related to several more or less relevant factors (comorbidities, less expected plasticity, or not necessarily founded medical fears). We can evoke the hypothesis of a major prognostic impact of "aggressive" and personalized therapeutic strategies able to reverse the poor prognosis of this factor.

**Pathology**. Our results indicate that histological oncotype did not influence OS between patients with astrocytomas, oligodendroglioma, or oligoastrocytoma. However, we present initial results, which, for the most part, correspond to the two previous WHO classifications. This heterogeneity probably limits the importance of our conclusions (reevaluation with the new WHO classification in progress).

**Contrast Enhancement**. Among the 339 patients, some contrast enhancement (CI) was present on initial MRI in 25.1% of cases. In agreement with other series, the presence of CI was not related to worse prognosis (17, 28). Even if the presence of CI is quite surprising, it is as part of all DLGG series, and it has been clearly shown that the presence of a patchy and faint enhancement did not have a worse prognostic (28). Accordingly, we decided to include these patients in the series. The presence of a more intense or nodular CI conventionally reflects the presence of malignant transformation. These cases were included in our series when the pathological data obtained just after the incriminated MRI were in favor of a real DLGG.

Therapeutic strategies are constantly discussed because of the long duration of the disease, knowledge evolution about effectiveness of therapies and better apprehension of patients' QoL. As far as possible, the therapeutic sequence was adapted to each patient. Therefore, a randomized control trial does not appear to be the most appropriate study design for this chronic pathology (29). In this part, we chose not to multiplicate log-rank tests to compare survival according to treatment but rather to highlight the main points that can help clinicians to treat better the patients while keeping their QoL. Among the remaining questions, we wonder about which chemotherapy we should propose as the first line and the best timing to introduce radiotherapy.

The impact of functional-based surgery in DLGG, first mentioned in the mid-1990s, is now well established (10, 30). Our updated results corroborate the main published data (31). A complete surgery performed by specialized teams remains the first treatment to offer the best prognosis while respecting patient’s QoL (30–42). We did not find any difference in OS between subtotal and partial surgery. This may be related to the fact that partial surgeries have residual postoperative volume close to subtotal surgeries.

In our series, the concept of “early treatment” means delivering at least one therapeutic modality within the 2 years following the radiological diagnosis. We did not find any significant difference between the two groups even if we note better results in the group "delayed treatment." This point could be explained by the selection of therapeutic strategies: the concept of "delayed treatment" was more easily proposed to young patients in excellent general condition with a small, not contrast-enhanced volume and a slow kinetic profile.

Regarding the type of first chemotherapy, we did not highlight any difference between patients who received PCV versus TMZ. In the subgroup analysis (initial volume <49 vs. >=49 cm^3^) even if no significant difference was found, PCV seems associated with a better survival especially when the initial volume is greater than 49 cm^3^. Clinicians preferred the use of TMZ because of less hematological or gonadal toxicities and less general status deterioration with the possibility of continuing a near-normal life during therapeutic periods (43, 44). For patients with initial volumes greater than 49 cm^3^ (related to poor prognosis), we nevertheless wonder about a switch of strategy and a return to PCV. The impact of PCV on OS was reported previously (15) (median OS of 13.3 years for the RT+CT group vs. 7.8 years for RT, *p*=0.003) even though a PCV arm is lacking in this trial. Otherwise, two phase II studies reported that patients with 1p/19q codeletion receiving adjuvant TMZ had a high rate of radiographic stability and favorable OS allowing to delay radiotherapy in a significant number of patients (45, 46).

In our series, the association PCV + RT is associated with long survivals with a median OS close to 20 years. Nevertheless, several studies show that the association PCV+RT has a significant toxicity (47, 48). Moreover, we do not have precise data concerning the long-term outcome of patients treated with PCV+RT in regards on cognition and QoL (49, 50). As long as these data remain inaccessible, it seems difficult to systematically propose this strategy even for high-risk patients.

In our series, 167 patients received radiotherapy during their illness. We did not find a difference in OS between patients who received early radiotherapy from those who received delayed radiotherapy. Our results are in agreement with the only randomized control trial that compared the OS of early versus delayed radiotherapy (20) and the only one to be included in the meta-analysis (51), which has the same conclusions as ours. Our analysis is also consistent with a recent retrospective study on the U.S. National Cancer Database comparing the OS of patients who received postoperative radiation and those who did not, in which the multivariable analysis of the radiation group was associated with worse OS (HR 2.06, *p* < 0.001) (52). Nevertheless, in our series, the group of patients treated with radiation after malignant transformation had clearly the worst prognostic. It would seem, *in fine*, that the effect of radiotherapy performed at the low-grade stage is the same regardless of the time of completion (early or late), whereas early radiotherapy could affect the QoL via the cognitive toxicity. To the contrary, delaying the timing of radiotherapy too much (after the malignant transformation) is associated with worse survival.

The current study has some limitations and biases. First, the data lacks pathology review, and some tumors might have been misclassified. A pathological revision using the 2016 WHO classification is in progress. Moreover, there was a lot of unavailable biomolecular data. During the revision, according to the available material, we supplement our data with molecular parameters (primarily 1p19q and IDH status). Despite the retained precautions to avoid confounding factors, this study is not a trial. The choice of each treatment was made at an individual level by integrating many parameters and always after the medical and surgical team’s discussion with the patient and his or her entourage. Therefore, the interpretation of prognosis factors related to the oncological decision is a delicate work. Their statistical significance is probably due to the multiple adapted lines of therapy over time. Nevertheless, despite these issues, the results of these personalized treatments were revealed to be equal to the superior estimated OS than previous reports while always keeping in mind the preservation of QoL. Finally, one of the main shortcomings is probably the lack of longitudinally QoL data. To complete the results of the impact of radiotherapy, especially its timing, as well as the importance of functional-based surgery, the patients’ QoL is among the most relevant questions and should be studied with more acuity in future work.

## Conclusion

We report a large single-center series of DLGG collected over 35 years. Our results confirm the major importance of the KPS score and the volume of the tumor at time of diagnosis. We highlight the favorable impact of surgery on survival, especially when resection is complete, and an identical impact of radiotherapy whatever the timing (early or late in the low-grade course) but ideally before malignant transformation. Personalized treatment, despite difficulties induced by the multiple possibilities, seems to offer the best compromise between a long survival and the preservation of the QoL.

## Data Availability Statement

The raw data supporting the conclusions of this article will be made available by the authors, without undue reservation.

## Author Contributions

The author contributions are as follows: Design and conceptualized study: TO, MB, HD, FR, LT. Data collection and analysis: TO, MB, HD, FR, LT. All authors contributed to the article and approved the submitted version.

## Conflict of Interest

The authors declare that the research was conducted in the absence of any commercial or financial relationships that could be construed as a potential conflict of interest.

## References

[1] Duffau H Toward an “active” cognitive assessment in patients with diffuse low-grade glioma. World Neurosurg (2014) 82(1-2):e129–31. 10.1016/j.wneu.2014.03.023 24636936

[2] Duffau H The challenge to remove diffuse low-grade gliomas while preserving brain functions. Acta Neurochir (Wien) (2012) 154(4):569–74. 10.1007/s00701-012-1275-7 22278663

[3] Klein M Duffau H De Witt Hamer PC Cognition and resective surgery for diffuse infiltrative glioma: an overview. J Neurooncol (2012) 108(2):309–18. 10.1007/s11060-012-0811-x PMC3351615 22362370

[4] Mandonnet E Duffau H An attempt to conceptualize the individual onco-functional balance: Why a standardized treatment is an illusion for diffuse low-grade glioma patients. Crit Rev Oncol Hematol (2018) 122:83–91. 10.1016/j.critrevonc.2017.12.008 29458793

[5] Duffau H Taillandier L New concepts in the management of diffuse low-grade glioma: Proposal of a multistage and individualized therapeutic approach. Neuro-Oncol (2015) 17(3):332–42. 10.1093/neuonc/nou153 PMC4483091 25087230

[6] Ryken TC Parney I Buatti J Kalkanis SN Olson JJ The role of radiotherapy in the management of patients with diffuse low grade glioma: A systematic review and evidence-based clinical practice guideline. J Neurooncol (2015) 125(3):551–83. 10.1007/s11060-015-1948-1 26530266

[7] Sarkiss CA Germano IM Machine Learning in Neuro-Oncology: Can Data Analysis from 5,346 Patients Change Decision Making Paradigms? World Neurosurg (2019) 124:287–94. 10.1016/j.wneu.2019.01.046 30684706

[8] Berger MS Deliganis AV Dobbins J Keles GE The effect of extent of resection on recurrence in patients with low grade cerebral hemisphere gliomas. Cancer (1994) 74(6):1784–91. 10.1002/1097-0142(19940915)74:6<1784::AID-CNCR2820740622>3.0.CO;2-D 8082081

[9] Pallud J Taillandier L Capelle L Fontaine D Peyre M Ducray F Quantitative morphological magnetic resonance imaging follow-up of low-grade glioma: a plea for systematic measurement of growth rates. Neurosurgery (2012) 71(3):729–39; discussion 739–740. 10.1227/NEU.0b013e31826213de 22668885

[10] Ben Abdallah M Blonski M Wantz-Mezieres S Gaudeau Y Taillandier L Moureaux J-M Statistical evaluation of manual segmentation of a diffuse low-grade glioma MRI dataset. Conf Proc IEEE Eng Med Biol Soc (2016) 2016:4403–6. 10.1109/EMBC.2016.7591703 28269254

[11] Kleihues P Burger PC Scheithauer BW The new WHO classification of brain tumours. Brain Pathol (1993) 3(3):255–68. 10.1111/j.1750-3639.1993.tb00752.x 8293185

[12] Louis DN Ohgaki H Wiestler OD Cavenee WK Burger PC Jouvet A The 2007 WHO classification of tumours of the central nervous system. Acta Neuropathol (2007) 114(2):97–109. 10.1007/s00401-007-0243-4 17618441 PMC1929165

[13] Louis DN Perry A Reifenberger G von Deimling A Figarella-Branger D Kavenee KW The 2016 World Health Organization Classification of Tumors of the Central Nervous System: a summary. Acta Neuropathol (2016) 131(6):803–20. 10.1007/s00401-016-1545-1 27157931

[14] Johannesen TB Langmark F Lote K Progress in long-term survival in adult patients with supratentorial low-grade gliomas: a population-based study of 993 patients in whom tumors were diagnosed between 1970 and 1993. J Neurosurg (2003) 99(5):854–62. 10.3171/jns.2003.99.5.0854 14609165

[15] Buckner JC Shaw EG Pugh SL Chakravarti A Gilbert MR Barger GR Radiation plus Procarbazine, CCNU, and Vincristine in Low-Grade Glioma. New Engl J Med (2016) 374(14):1344–55. 10.1056/NEJMoa1500925 PMC5170873 27050206

[16] Jakola AS Skjulsvik AJ Myrmel KS Sjåvik K Unsgard G Torp SH Surgical resection versus watchful waiting in low-grade gliomas. Ann Oncol (2017) 28(8):1942–8. 10.1093/annonc/mdx230 PMC5834105 28475680

[17] Garcia CR Slone SA Pittman T St Clair WH Lightner DD Villano JL Comprehensive evaluation of treatment and outcomes of low-grade diffuse gliomas. PloS One (2018) 13(9):e0203639. 10.1371/journal.pone.0203639 30235224 PMC6147430

[18] Youland RS Brown PD Giannini C Parney IF Uhm JH Laack NN Adult low-grade glioma: 19-year experience at a single institution. Am J Clin Oncol (2013) 36(6):612–9. 10.1097/COC.0b013e31825d580a PMC4361933 22892428

[19] Baumert BG Hegi ME van den Bent MJ von Deimling A Gorlia T Hoang-Xuan K Temozolomide chemotherapy versus radiotherapy in high-risk low-grade glioma (EORTC 22033-26033): a randomised, open-label, phase 3 intergroup study. Lancet Oncol (2016) 17(11):1521–32. 10.1016/S1470-2045(16)30313-8 PMC5124485 27686946

[20] van den Bent MJ Afra D de Witte O Schraub S Hoang-Xuan K Malmström P-O Long-term efficacy of early versus delayed radiotherapy for low-grade astrocytoma and oligodendroglioma in adults: the EORTC 22845 randomised trial. Lancet (2005) 366(9490):985–90. 10.1016/S0140-6736(05)67070-5 16168780

[21] Kim Y-H Nobusawa S Mittelbronn M Paulus W Brokinkel B Keyvani K Molecular classification of low-grade diffuse gliomas. Am J Pathol (2010) 177(6):2708–14. 10.2353/ajpath.2010.100680 PMC2993282 21075857

[22] Schomas DA Laack NNI Rao RD Meyer FB Shaw EG O’Neill BP Intracranial low-grade gliomas in adults: 30-year experience with long-term follow-up at Mayo Clinic. Neuro-Oncol (2009) 11(4):437–45. 10.1215/15228517-2008-102 PMC2743224 19018039

[23] Pignatti F van den Bent M Curran D Debruyne C Sylvester R Therasse P Prognostic factors for survival in adult patients with cerebral low-grade glioma. J Clin Oncol (2002) 20(8):2076–84. 10.1200/JCO.2002.08.121 11956268

[24] Majchrzak K Kaspera W Bobek-Billewicz B Hebda A Stasik-Pres G Majchrzak H The assessment of prognostic factors in surgical treatment of low-grade gliomas: a prospective study. Clin Neurol Neurosurg (2012) 114(8):1135–44. 10.1016/j.clineuro.2012.02.054 22425370

[25] Daniels TB Brown PD Felten SJ Wu W Buckner JC Arusell RM Validation of EORTC prognostic factors for adults with low-grade glioma: a report using intergroup 86-72-51. Int J Radiat Oncol Biol Phys (2011) 81(1):218–24. 10.1016/j.ijrobp.2010.05.003 PMC3151343 21549518

[26] Ius T Angelini E Thiebaut de Schotten M Mandonnet E Duffau H Evidence for potentials and limitations of brain plasticity using an atlas of functional resectability of WHO grade II gliomas: towards a “minimal common brain.” Neuroimage (2011) 56(3):992–1000. 10.1016/j.neuroimage.2011.03.022 21414413

[27] Schomas DA Laack NN Brown PD Low-grade gliomas in older patients: long-term follow-up from Mayo Clinic. Cancer (2009) 115(17):3969–78. 10.1002/cncr.24444 PMC2789453 19536875

[28] Pallud J Capelle L Taillandier L Fontaine D Mandonnet E Guillevin R Prognostic significance of imaging contrast enhancement for WHO grade II gliomas. Neuro-Oncol (2009) 11(2):176–82. 10.1215/15228517-2008-066 PMC2718989 18697954

[29] Duffau H Paradoxes of evidence-based medicine in lower-grade glioma: To treat the tumor or the patient? Neurology (2018) 91(14):657–62. 10.1212/WNL.0000000000006288 30158156

[30] Hervey-Jumper SL Berger MS Technical nuances of awake brain tumor surgery and the role of maximum safe resection. J Neurosurg Sci (2015) 59(4):351–60. 26394241

[31] Aghi MK Nahed BV Sloan AE Ryken TC Kalkanis SN Olson JJ The role of surgery in the management of patients with diffuse low grade glioma: A systematic review and evidence-based clinical practice guideline. J Neurooncol (2015) 125(3):503–30. 10.1007/s11060-015-1867-1 26530265

[32] Yang K Nath S Koziarz A Badhiwala JH Ghayur H Sourour M Biopsy Versus Subtotal Versus Gross Total Resection in Patients with Low-Grade Glioma: A Systematic Review and Meta-Analysis. World Neurosurg (2018) 120:e762–75. 10.1016/j.wneu.2018.08.163 30172972

[33] Berger MS Rostomily RC Low grade gliomas: functional mapping resection strategies, extent of resection, and outcome. J Neurooncol (1997) 34(1):85–101. 10.1023/a:1005715405413 9210055

[34] Xia L Fang C Chen G Sun C Relationship between the extent of resection and the survival of patients with low-grade gliomas: a systematic review and meta-analysis. BMC Cancer (2018) 18(1):48. 10.1186/s12885-017-3909-x 29306321 PMC5756328

[35] Duffau H Lopes M Arthuis F Bitar A Sichez JP Van Effenterre R Contribution of intraoperative electrical stimulations in surgery of low grade gliomas: a comparative study between two series without (1985-96) and with (1996-2003) functional mapping in the same institution. J Neurol Neurosurg Psychiatry (2005) 76(6):845–51. 10.1136/jnnp.2004.048520 PMC1739650 15897509

[36] De Witt Hamer PC Robles SG Zwinderman AH Duffau H Berger MS Impact of intraoperative stimulation brain mapping on glioma surgery outcome: a meta-analysis. J Clin Oncol (2012) 30(20):2559–65. 10.1200/JCO.2011.38.4818 22529254

[37] Keles GE Lamborn KR Berger MS Low-grade hemispheric gliomas in adults: a critical review of extent of resection as a factor influencing outcome. J Neurosurg (2001) 95(5):735–45. 10.3171/jns.2001.95.5.0735 11702861

[38] Duffau H Long-term outcomes after supratotal resection of diffuse low-grade gliomas: a consecutive series with 11-year follow-up. Acta Neurochir (Wien) (2016) 158(1):51–8. 10.1007/s00701-015-2621-3 26530708

[39] Bady P Kurscheid S Delorenzi M Gorlia T Van den Bent MJ Hoang-Xuan K The DNA methylome of DDR genes and benefit from RT or TMZ in IDH mutant low-grade glioma treated in EORTC 22033. Acta Neuropathol (2018) 135(4):601–15. 10.1007/s00401-018-1810-6 PMC5978935 29368212

[40] Carstam L Smits A Milos P Corell A Henriksson R Bartek J Jr Neurosurgical patterns of care for diffuse low-grade gliomas in Sweden between 2005 and 2015. Neurooncol Pract (2019) 6(2):124–33. 10.1093/nop/npy023 PMC6440530 30949360

[41] Turkoglu E Gurer B Sanli AM Dolgun H Gurses L Oral NA Clinical outcome of surgically treated low-grade gliomas: a retrospective analysis of a single institute. Clin Neurol Neurosurg (2013) 115(12):2508–13. 10.1016/j.clineuro.2013.10.010 24225484

[42] Hervey-Jumper SL Berger MS Role of surgical resection in low- and high-grade gliomas. Curr Treat Options Neurol (2014) 16(4):284. 10.1007/s11940-014-0284-7 24595756

[43] Trinh VA Patel SP Hwu W-J The safety of temozolomide in the treatment of malignancies. Expert Opin Drug Saf (2009) 8(4):493–9. 10.1517/14740330902918281 19435405

[44] Hafazalla K Sahgal A Jaja B Perry JR Das S Procarbazine, CCNU and vincristine (PCV) versus temozolomide chemotherapy for patients with low-grade glioma: a systematic review. Oncotarget (2018) 9(72):33623–33. 10.18632/oncotarget.25890 PMC6154749 30263090

[45] Wahl M Phillips JJ Molinaro AM Lin Y Perry A Haas-Kogan DA Chemotherapy for adult low-grade gliomas: clinical outcomes by molecular subtype in a phase II study of adjuvant temozolomide. Neuro-Oncology (2016) now176. 10.1093/neuonc/now176 PMC5464133 27571885

[46] Rudà R Pellerino A Pace A Carapella CA Dealis C Caroli M Efficacy of initial temozolomide for high-risk low grade gliomas in a phase II AINO (Italian Association for Neuro-Oncology) study: a post-hoc analysis within molecular subgroups of WHO 2016. J Neurooncol (2019) 145(1):115–23. 10.1007/s11060-019-03277-x 31556015

[47] Lecavalier-Barsoum M Quon H Abdulkarim B Adjuvant treatment of anaplastic oligodendrogliomas and oligoastrocytomas. Cochrane Database Syst Rev (2014) (5):CD007104. 10.1002/14651858.CD007104.pub2 PMC7388823 24833028

[48] Jutras G Bélanger K Letarte N Adam J-P Roberge D Lemieux B Procarbazine, lomustine and vincristine toxicity in low-grade gliomas. Curr Oncol (2018) 25(1):e33–9. 10.3747/co.25.3680 PMC5832288 29507493

[49] Olson JD Riedel E DeAngelis LM Long-term outcome of low-grade oligodendroglioma and mixed glioma. Neurology (2000) 54(7):1442–8. 10.1212/WNL.54.7.1442 10751254

[50] Lassman AB Procarbazine, lomustine and vincristine or temozolomide: which is the better regimen? CNS Oncol (2015) 4(5):341–6. 10.2217/cns.15.36 PMC6082332 26544062

[51] Sarmiento JM Venteicher AS Patil CG Early versus delayed postoperative radiotherapy for treatment of low-grade gliomas. Cochrane Database Syst Rev (2015) (6):CD009229. 10.1002/14651858.CD009229.pub2 PMC4506130 26118544

[52] Youssef I Lee A Garay EL Becker DJ Schreiber D Patterns of care and outcomes of postoperative radiation for low-grade gliomas in United States hospitals. J Clin Neurosci (2018) 58:124–9. 10.1016/j.jocn.2018.09.010 30287250

